# Characteristics and outcomes of patients with dyspnoea as the main symptom, assessed by prehospital emergency nurses- a retrospective observational study

**DOI:** 10.1186/s12873-020-00363-6

**Published:** 2020-08-28

**Authors:** Wivica Kauppi, Johan Herlitz, Carl Magnusson, Lina Palmér, Christer Axelsson

**Affiliations:** 1 PreHospen- Centre for Prehospital Research, Faculty of Caring, Work Life and Social Welfare, University of Borås, SE- 501 90 Borås, Sweden; 2 Faculty of Caring Science, Work Life and Social Welfare, University of Borås, Borås, Sweden; 3 Department of Molecular and Clinical Medicine, Sahlgrenska Academy, University of Gothenburg, Gothenburg, Sweden

**Keywords:** Dyspnoea, Epidemiology, Aetiology, Major incidents, Prevention, Emergency medical service, Prehospital emergency nurse, Ambulance

## Abstract

**Background:**

Dyspnoea (breathing difficulty) is among the most commonly cited reasons for contacting emergency medical services (EMSs). Dyspnoea is caused by several serious underlying medical conditions and, based on patients individual needs and complex illnesses or injuries, ambulance staff are independently responsible for advanced care provision. Few large-scale prehospital studies have reviewed patients with dyspnoea. This study aimed to describe the characteristics and final outcomes of patients whose main symptom was classified as dyspnoea by the prehospital emergency nurse (PEN).

**Methods:**

This retrospective observational study included patients aged > 16 years whose main symptom was dyspnoea. All the enrolled patients were assessed in the south-western part of Sweden by PENs during January and December, 2017. Of 7260 assignments (9% of all primary missions), 6354 fulfilled the inclusion criteria. Analysis was performed using descriptive statistics, and the tests used were odds ratios and Kaplan-Meier analysis.

**Results:**

The patients mean age was 73 years, and approximately 56% were women. More than 400 different final diagnostic codes (International Statistical Classification of Diseases and Related Health Problems [ICD]-10th edition) were observed, and 11% of the ICD-10 codes denoted time-critical conditions. The three most commonly observed aetiologies were chronic obstructive pulmonary disease (20.4%), pulmonary infection (17.1%), and heart failure (15%). The comorbidity values were high, with 84.4% having previously experienced dyspnoea. The overall 30-day mortality was 11.1%. More than half called EMSs more than 50 h after symptom onset.

**Conclusions:**

Among patients assessed by PENs due to dyspnoea as the main symptom there were more than 400 different final diagnoses, of which 11% were regarded as time-critical. These patients had a severe comorbidity and 11% died within the first 30 days.

## Background

Dyspnoea (breathing difficulty) is among the most commonly cited reasons for contacting emergency medical services (EMSs) [1]. The American Thoracic Society defines dyspnoea as “a subjective experience of breathing discomfort that consists of qualitatively distinct sensations that vary in intensity” [2]. It manifests itself in various ways such as shortness of breath, air hunger, and chest tightness [3]. Dyspnoea is caused by several medical conditions [4, 5], and respiratory failure is among the most severe time-critical conditions for which the provision of immediate prehospital care by EMSs can have a highly valuable impact [6].

In Sweden, prehospital emergency nurses (PENs) have an important role in the assessment and triaging of dyspnoea patients at the scene. The treatment of dyspnoea begins with the management of the underlying condition, due to which the diagnosis of acute dyspnoea is crucial in EMS settings to ensure the provision of appropriate treatment and care [7]. Patients with dyspnoea constitute a complex group, as the presence of additional health problems makes it difficult to identify the underlying cause of the condition. In addition to the physiological perspective, severe anxiety and fear are observed commonly in connection with dyspnoea. This denotes that PENs have the responsibility of providing care aimed at increasing patients strength and focusing on their existential needs [8–10]. Previous studies have described the epidemiology and outcomes of patients with dyspnoea who are transported by EMSs to emergency departments (EDs) in Denmark, Australia/New Zealand and the United States [8, 11, 12]. However, few large-scale EMS studies have been conducted in such settings till date. In the present study, we aimed to describe the characteristics and final outcomes, overall and in relation to sex of patients receiving care from PENs and whose main symptom was dyspnoea.

## Methods

### Design

A quantitative, exploratory, descriptive design was employed based on a consecutive retrospective review of EMS and hospital records. The study included all patients over a one-year time-period who called the emergency number (112) in Sweden, had an ambulance dispatched and were assessed by the prehospital emergency nurses (PENs) with the main symptom of dyspnoea.

### Settings

#### Population

**Fig. 1:**
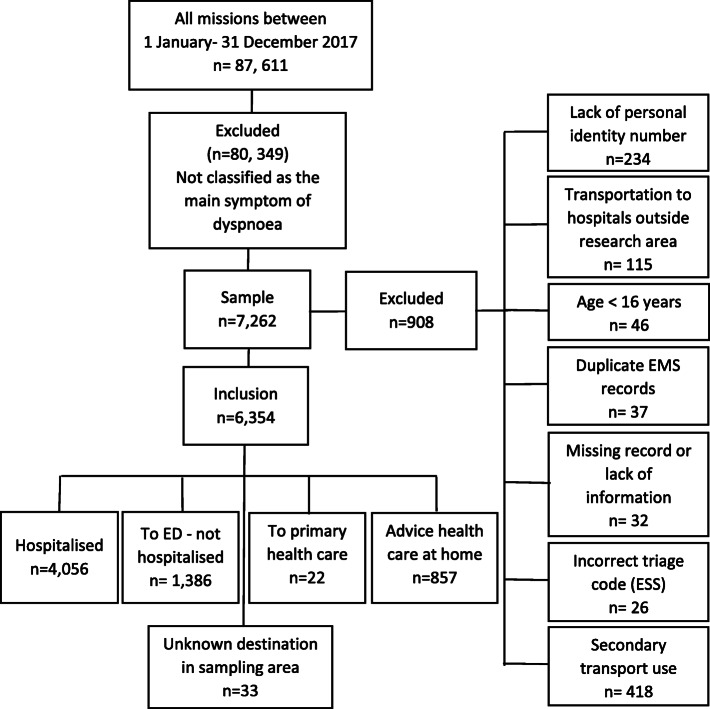
Flow chart of the studied patients, assessed as the main symptom of dyspnoea

The study was conducted within two EMS organisations, which together include 16 ambulance stations in the south-western part of Sweden. The two EMS organisations cover a combined area of 7400 km^2^, with a population of 962,000 inhabitants in urban, suburban, and rural areas. During 2017, the two EMS organisations had 123,614 ambulance missions with a priority level of 1 to 3. Of these, 87,611 missions involved an initial patient assessment defined as the primary mission (Fig. 1).

#### Competence of Swedish ambulances, and the associated guidelines and triage system

Since 2005, all ambulances in Sweden have at least one registered nurse and one ambulance technician [13]. A majority of all such nurses have completed a three-year nursing course followed by a one-year Master’s course focusing on prehospital emergency care and have the professional title ‘PEN’. PENs provide assessment and treatment according to national and local guidelines.

In order to assess and prioritise patients degree of prehospital care required, PENs perform triage classification according to the Rapid Emergency Triage and Treatment System for adults (RETTS- A) [14]. RETTS-A codes are divided into the following types: 1) vital signs (VS) and 2) 53 emergency signs and symptoms (ESS) codes [see Additional file 1: Red and orange VS and ESS code 04, dyspnoea]. Both types allow for the allocation of patients separately to a severity/triage level in which the highest level is used for the final assessment. The severity/triage level is assigned one of five different colours (red, orange, yellow, green, and blue) according to the time from assessment to the time a patient must be seen by a physician. Red and orange indicate the most urgent cases whereas yellow and green denote the absence of individual medical risk even if patients are made to wait to undergo physician assessment. The colour blue is not used in the prehospital triage. In ESS code 04 (dyspnoea as the main symptom), it is mandatory for the PENs to evaluate ECG in all patients. In addition of all prehospital severe ill patients, it is also mandatory to evaluate the P-Glucose level.

### Inclusion and exclusion criteria

The inclusion criteria were as follows: 1) a primary mission in which a patient (or e.g. a family member that represents the patient) calls the dispatch centre, an ambulance is dispatched and 2) the assignment of an ESS code of 04 (dyspnoea) is made by the PEN. The exclusion criteria were as follows: 1) lack of a personal identity number, 2) transportation to hospitals outside the research area, 3) age < 16 years, 4) duplicate EMS records, 5) missing record or lack of information, 6) incorrect triage (ESS), and 7) secondary transport use. A total of 7260 patients were initially identified from the sample (9% of all assignments). After a manual review, 908 patients were excluded due to various exclusion criteria, and a total of 6354 patients were finally included in the study (Fig. 1). Among the 6354 patients, a total of 3665 patients had one EMS contact during the study period and 2689 patients had multiple contacts. A total of 4587 unique patients were included in the survival analysis of which 922 patients were randomly selected from the patients with multiple contacts.

### Endpoints

The primary endpoint was the aetiology according to the final diagnosis. Secondary endpoints were comorbidity according to patients previous history and the 30- day mortality. Tertiary endpoints were the patients delay time from symptom onset until calling for EMS and clinical findings on arrival of PENs.

### Data collection

The patients in this study were consecutively included by assignment through an EMS record database (Ambulink) and followed-up by hospital records (Melior) according to the directions for inclusion and exclusion. Ambulink contains the RETTS-A triage classification and Melior contains the International Classification of Diseases (ICD) code. Medical history and final diagnoses were categorised using the ICD-10 which comprises 22 chapters (I–XXII). In all the ambulances, only 12 lead ECG was used. In order to achieve the best possible data quality, data on ECG interpretations were collected first from prehospital records, second from ED notes, and third, in cases in which no interpretation was documented but an ECG was recorded, by the author (WK). ECG deviations comprise atrial fibrillation/flutter, ST-elevation, ST-depression, T-wave inversion, and left bundle branch block. Other deviations include AV-block I, II, III, ectopic atrial rhythm/tachycardia, and ventricular tachycardia. P-glucose levels were measured through a capillary test performed by the PEN, and a value ≥9.5 mmol/l was defined as high [15].

Time-critical conditions are defined as conditions where prompt management and medical interventions are crucial to avoid severe complications and early death [16, 17].

These were defined by our research group consisting of a cardiologist and number of PENs based on the available literature and clinical experience. This definition has been previously described [16]. However, a few further diagnoses (e.g. acute respiratory failure with hypercapnia or hypoxia, ketoacidosis and lactacidosis) fulfilling the above criteria appeared in the analyses of the present study cohort and have therefore been added.

### Statistics

**Fig. 2:**
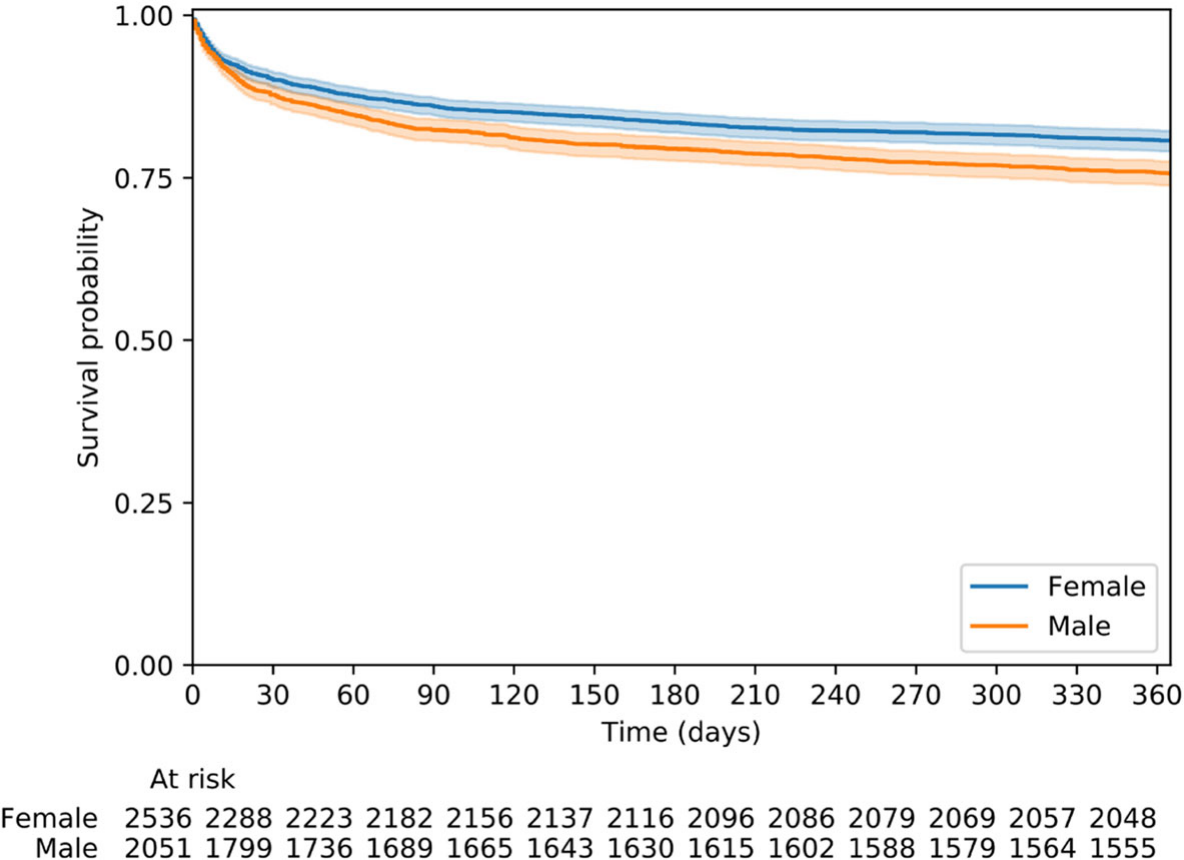
Kaplan-Meier survival plot of patients assessed by the PEN as the main symptom of dyspnoea and stratified by women and men

In the tables, results are presented as number (percentage), median (25th, 75th percentiles), or mean (standard deviation). For two-group comparisons, crude odds ratios with their correspondent confidence intervals were calculated. A Kaplan-Meier survival curve stratified by sex was plotted (Fig. 2), while the lifelines survival function was used to report differences by sex for 30-day mortality. All tests are two-sided, and due to the number of tests performed, *p*-values < 0.01 were considered significant. Confidence intervals computed at the 99% level were used. Data processing and statistical analyses were performed using SPSS version 24 (Armonk, NY, USA: IBM Corp.) and Python version 3.7 (Python software foundation) with lifelines package (Cameron Davidson-Pilon, Jonas Kalderstam, Paul Zivich, et al. CamDavidsonPilon/lifelines: v0.23.9. 2020).

## Results

Totally, 7262 EMS missions fulfilled the inclusion criteria, and 908 cases (13%) were excluded due to reasons shown in Fig. 1. Overall, patients with the ESS code 04 (dyspnoea as the main symptom) conformed 9% of all primary EMS missions and more than 60% of those patients were hospitalised (Fig. 1).

### Age, sex, and previous history

The patients’ mean age was 73 years, and 56% of them were women. A majority (84.4%) had previously experienced dyspnoea. A large proportion had a history of cardiovascular disease including hypertension (46.5%), heart failure (30%), atrial fibrillation (29%), ischemic heart disease (26%). Furthermore, 47.5% had a history of pulmonary disease, 18.8% had diabetes and 19.4% had cancer. Men had a higher prevalence of previous heart disease, diabetes and renal disease whereas women had a higher prevalence of previous pulmonary disease, system disease and psychiatric disease (Table 1).

**Table 1:** Age, sex and previous history

	All patients	Women	Men		Missing	
n	6354	3538	2816	All	Women	Men
**Age - years**						
Mean (±SD)	72.9 (17.5)	73.3 (18.1)	72.5 (16.6)			
Median (25th,75th percentile)	77 (66,86)	78 (67,86)	76 (66,85)			
**Previous history - n(%)**						
Dyspnoea	4971 (84.4)	2766 (84.0)	1.07 [0.89,1.29]^a^	467	247	220
Pulmonary disease^b^	2966 (47.5)	1762 (50.6)	0.76 [0.66,0.86]	116	59	57
Hypertension	2948 (46.5)	1718 (48.6)	0.82 [0.72,0.94]	8	5	3
Heart failure	1894 (30.0)	961 (27.4)	1.32 [1.15,1.52]	38	27	11
Atrial fibrillation	1838 (29.0)	945 (26.8)	1.27 [1.10,1.47]	14	8	6
Ischaemic heart disease	1642 (26.0)	785 (22.3)	1.54 [1.33,1.79]	40	16	24
Psychiatric disorder^c^	1337 (21.2)	862 (24.5)	0.63 [0.53,0.74]	34	20	14
Cancer	1226 (19.4)	669 (19.0)	1.06 [0.90,1.25]	23	15	8
Diabetes	1190 (18.8)	550 (15.6)	1.60 [1.35,1.89]	12	7	5
Renal disease	759 (12.0)	310 (8.8)	1.97 [1.61,2.42]	12	8	4
System disease	393 (6.2)	286 (8.1)	0.45 [0.33,0.61]	17	12	5
Other disease^d^	4412 (69.5)	2458 (69.6)	1.00 [0.87,1.15]	8	4	4

^a^Odds ratio and 99% - confidence interval

^b^Chronic obstructive pulmonary disease, asthma bronchiale, other pulmonary disease

^c^Panic disorder, anxiety disorder, depression, bipolar disease, schizofrenia, other psychiatric disorder

^d^Other disease: e.g. peripheral vascular diseases, other heart conditions, previous pulmonary embolism

### Symptoms and clinical findings

In all, 23.1% of the patients had pain, 1.2% had syncope, and 1.6% showed alcohol/drug-related issues. Abnormal vital parameters were seen with the following rates (according to the RETTS-A): respiratory rate < 8/min or > 25/min (48.7%), oxygen saturation < 90% (35.2%), heart rate < 40/min or > 120/min (11.5%), systolic blood pressure < 90 mmHg (1.8%), body temperature < 35 or > 41 degrees (0.5%), and degree of consciousness > RLS 2 (1.6%). An ECG was recorded in 74% of the cases; 70.8% of them showed a sinus rhythm, whereas 22.8% showed atrial fibrillation/flutter. Signs of possible myocardial ischemia were revealed as ST-elevation (2.5%), ST-depression (13.9%), T-wave inversion (9.5%), and left bundle branch block (6%). P-glucose levels were measured in 31.3% of the cases, of whom 37.6% showed elevated values. Men had more often deviation of the systolic blood pressure and atrial fibrillation than women (Table 2).

**Table 2:** Symptoms and clinical findings

	All patients	Women	Men		Missing	
n	6354	3538	2816	All	Women	Men
**Time interval - median hh:mm**^a^						
Symptom onset - call for EMS	51:08 (3:57,145:02)	50:58 [47:49,55:18]	51:11 [47:47,55:59]	438	245	193
**Symptoms - n(%)**						
Pain	1402 (23.1)	806 (23.9)	0.91 [0.78,1.07]^b^	292	162	130
If yes, VAS median^a^	4 (2,6)	3 [2,4]	4 [3,5]	1149	660	489
Syncope	77 (1.2)	47 (1.3)	0.80 [0.44,1.47]	24	14	10
Affected by alcohol, drugs	103 (1.6)	46 (1.3)	1.57 [0.94,2.63]	36	17	19
**Vital signs; first recording – median**^**c**^						
Respiratory rate (breaths/min)	25 (20,32) 48.7	25 (20,32) 49.1	0.96 [0.84,1.01]	137	70	67
Oxygen saturation (%)	93 (86,97) 35.2	93 (85,97) 36.0	0.92 [0.81,1.06]	120	55	65
Heart rate (beats/min)	92 (80,110) 11.5	94 (80,110) 11.7	0.95 [0.77,1.17]	122	58	64
Systolic blood pressure (mmHg)	140 (120,160) 1.8	140 (120,160) 1.2	2.15 [1.30,3.57]	236	132	104
Diastolic blood pressure (mmHg)	80 (70,90) 0.3	80 (70,90) 0.3	1.26 [0.37,4.25]	870	482	388
Body temperature (°C)	37.0 (36.6,37.4) 0.5	37.0 (36.6,37.5) 0.5	1.34 [0.55,3.29]	234	128	106
Degree of consciousness (RLS)	1 (1,1) 1.6	1 (1,1) 1.7	0.91 [0.51,1.60]	1110	616	494
**ECG recorded in ambulance - n(%)**	4693 (74.0)	2540 (71.9)	1.28 [1.10,1.49]	8	4	4
**ECG rhythm - n(%)**^**d**^				873	512	361
Sinus rythm	3882 (70.8)	2257 (74.6)	0.67 [0.57,0.79]			
Atrial fibrillation/flutter	1249 (22.8)	648 (21.4)	1.19 [1.01,1.41]			
Other rythm^e^	350 (6.4)	121 (4.0)	2.47 [1.83,3.33]			
**ECG pattern - n(%)**^**d**^				957	569	388
ST-elevation	137 (2.5)	67 (2.3)	1.29 [0.82,2.01]			
ST-depression	748 (13.9)	420 (14.1)	0.95 [0.77,1.16]			
T-wave inversion	511 (9.5)	270 (9.1)	1.10 [0.87,1.40]			
Left bundle branch block	325 (6.0)	181 (6.1)	0.97 [0.72,1.31]			
**Blood Glucose measured - n(%)**	1991 (31.3)	1034 (29.2)	1.25 [1.08,1.43]			
Blood glucose elevation ≥9.5 mmol/l	749 (37.6)	402 (38.9)	0.89 [0.70,1.14]			

^a^All patients category denoted with 25th and 75th percentiles, women and men with 99% - confidence intervals;VAS: visual analogue scale

^b^Odds ratio and 99% - confidence interval

^**c**^All patients and women category denoted with median (25th,75th percentiles) and percetage of deviating vital signs. Odds ratio and 99%-confidence intervals calculated on number of deviating vital signs

Deviating vital signs: Respiratory rate < 8 or > 25 breaths/min; Oxygen saturation < 90%; Heart rate < 40/min or > 120/min; Systolic blood pressure < 90 mm/Hg; Diastolic blood pressure > 140 mm/hg; Body temperature (°C) < 35 or > 41; Reaction level scale (RLS) > 2

^**d**^Electrocardiogram (ECG) rhythm and pattern recorded in Ambulance or in the ED within 4 h

^e^Pacemaker rhythm, Av-block I,II,III, Ectopical atrial rhythm/takycardia, Idioventricular rhytm, Junctional rhythm, Pulseless electrical activity, Ventricular takycardia

### Distribution of patients across the 22 major ICD-10 code groups

The three most commonly observed disease groups were: diseases of the respiratory system (49.1%); diseases of the circulatory system (23.1%); and symptoms, signs, and abnormal clinical findings not elsewhere classified (13.4%). Women had more often a respiratory disease (Table 3).

**Table 3:** Distribution of patients across the 23 major ICD-10 code groups

	All patients^a^	Women	Men
Chapters - n(%)	6354	3538	2816
I Certain infections and parasites diseases A00 – B99	161 (3.0)	75 (2.6)	1.40 [0.92,2.10]^b^
II Neoplasm C00 – D48	131 (2.5)	62 (2.1)	1.35 [0.85,2.12]
III Diseases of the blood and blood forming organs and certain diseases involving the immuno-mechanisms D50 – D89	34 (0.6)	16 (0.6)	1.35 [0.56,3.29]
IV Endocrine, nutritional and metabolic diseases E00 – E90	42 (0.8)	25 (0.9)	0.82 [0.36,1.84]
V Mental and behavioural disorders F00 – F99	91 (1.7)	50 (1.7)	0.98 [0.57,1.70]
VI Diseases of the nervous system G00 – G99	29 (0.5)	17 (0.6)	0.85 [0.32,2.24]
VII Diseases of the eye and adnexa H00 – H59	0 (0.0)	0 (0.0)	0 [0,0]
VIII Diseases of the eye and mastoid process H60 – H95	0 (0.0)	0 (0.0)	0 [0,0]
IX Diseases of the circulatory system I00 – I99	1230 (23.1)	647 (22.3)	1.11 [0.94,1.31]
X Diseases of the respiratory system J00 – J99	2615 (49.1)	1476 (50.8)	0.86 [0.75,0.99]
XI Diseases of the digestive system K00 – K93	65 (1.2)	33 (1.1)	1.17 [0.61,2.22]
XII Diseases of the skin and subcutaneous tissue L00 – L99	6 (0.1)	3 (0.1)	1.20 [0.15,9,85]
XIII Diseases of the musculoskeletal tissue and connective tissue M00 – M99	51 (1.0)	37 (1.3)	0.45 [0.20,1.02]
XIV Diseases of the genitourinery system N00 – N99	69 (1.3)	30 (1.0)	1.57 [0.84,2.95]
XV Pregnancy, childbirth and puerperium O00 – O99	1 (< 0.1)	1 (< 0.1)	0 [0,0]
XVI Certain conditions originating from the perinatal period P00 – P96	0 (0.0)	0 (0.0)	0 [0,0]
XVII Congenital malformations, deformation and chromosomal malformations Q00 – Q99	1 (< 0.1)	0 (0.0)	0 [0,0]
XVIII Symptoms, signs and abnormal clinical findings, not elsewhere classified R00 – R99	711 (13.4)	382 (13.2)	1.04 [0.84,1.28]
XIX Injury, Poisoning and certain other consequences of external causes S00 – T98	33 (0.6)	23 (0.8)	0.52 [0.20,1.38]
XX External causes of morbidity and mortality V00 – V99	1 (< 0.1)	1 (< 0.1)	0 [0,0]
XXI External causes to disease and death Y01 – Y98	1 (< 0.1)	1 (< 0.1)	0 [0,0]
XXII Factors influencing health status and contact with health services Z00 – Z99	48 (0.9)	23 (0.8)	1.31 [0.62,2.76]
XXIII Codes for special purposes U00-U99	4 (0.1)	3 (0.1)	0.40 [0.20,7.84]

^a^Missing diagnosis in 1030 patients (women 633, men 397)

^b^Odds ratio and 99% - confidence interval

### Distribution of patients according to the final diagnosis (ICD-10 code) among groups more than 30 patients

The patients showed 473 different ICD-10 codes pointing to the primary aetiology. The three most commonly observed aetiologies were Chronic Obstructive Pulmonary Disease (COPD) (20.4%), pulmonary infection (17.1%), and heart failure (15%). Totally, 11% of the ICD-10 codes showed time-critical conditions. After combining these ICD-10 codes, the three most common conditions were cardiovascular disease, followed by infection and pulmonary disease. Women more often had pulmonary disease (Table 4).

**Table 4:** Distribution of patients according to final diagnosis (ICD-10 code) among groups more than 30 patients

	All patients^a^	Women	Men
Final diagnosis - n(%)	6354	3538	2816
**1. Cardiovascular disease**	1316 (24.7)	694 (23.9)	1.10 [0.94,1.30]^b^
a. Heart failure	801 (15.0)	402 (13.8)	399 (16.5)
b. Ischemic heart disease	154 (2.9)	70 (2.4)	84 (3.5)
c. Arrhythmia	142 (2.7)	87 (3.0)	55 (2.3)
d. Pulmonary embolism	123 (2.3)	81 (2.8)	42 (1.7)
**2. Pulmonary disease**	1311 (24.6)	780 (26.9)	0.77 [0.65,0.91]
a. Chronic obstructive pulmonary disease	1088 (20.4)	656 (22.6)	432 (17.9)
b. Asthma bronchiale	127 (2.4)	87 (3.0)	40 (1.7)
c. Other pulmonary diseases	94 (1.8)	37 (1.3)	57 (2.4)
**3. Infection**	1172 (22.0)	607 (20.9)	1.15 [0.97,1.37]
a. Pulmonary	912 (17.1)	469 (16.1)	443 (18.3)
b. Sepsis	97 (1.8)	43 (1.5)	54 (2.2)
c. Ear, nose and throat and upper airways	81 (1.5)	52 (1.8)	29 (1.2)
d. Other infection	74 (1.4)	40 (1.4)	34 (1.4)
**4. Symptom diagnosis**	662 (12.4)	353 (12.2)	1.06 [0.85,1.31]
**5. Cancer**	138 (2.6)	66 (2.3)	1.32 [0.85,2.01]
a. Pulmonary	89 (1.7)	49 (1.7)	40 (1.7)
b. Other localisation	31 (0.6)	13 (0.4)	18 (0.7)
**6. Respiratory insufficiency**	129 (2.4)	79 (2.7)	0.76 [0.47,1.21]
**7. Psychiatric disorder**	90 (1.7)	49 (1.7)	1.01 [0.58,1.74]
**9. Urinary disease**	69 (1.3)	30 (1.0)	1.57 [0.84,2.95]
**10. Pleura Disease**	54 (1.0)	32 (1.1)	0.82 [0.40,1.69]
**11. Muscle skeletal pain**	44 (0.8)	32 (1.1)	0.45 [0.19,1.07]
**12. Organ failure**	39 (0.7)	23 (0.8)	0.84 [0.36,1.95]
**13. Gastrointestinal disease**	33 (0.6)	18 (0.6)	1.00 [0.41,2.47]
**14. Anaemia**	33 (0.6)	15 (0.5)	1.44 [0.59,3.56]

^a^Missing diagnosis in 1030 patients (women 633, men 397)

^b^Odds ratio with 99% - confidence interval, subcategories with percentage

### Mortality

The overall 30-day, and 1-year mortality values were 11.1, and 21.5%, respectively. Men had a higher 1-year mortality than women (*p* < 0.005)(Fig. 2). The three most common final diagnoses among all patients who died within 30 days were pneumonia, followed by heart failure and COPD with acute exacerbation. The three most common final diagnoses among patients with time-critical conditions who died within 30 days were stroke, sepsis followed by acute respiratory insufficiency.

## Discussion

In this study, dyspnoea was classified as the main symptom in approximately 9% of all the EMS-assigned patients, similar to previous findings [1, 9, 12]. The most novel information was that among patients with dyspnoea there were more than 400 different final diagnoses. (ICD-10 codes) and 11% of them were assessed as time critical. Many of the patients had a severe comorbidity and 11% were dead within 30 days. Despite this, only half of the patients dialled 112 within 50 h after onset of symptoms.

The three most commonly observed disease groups were those of the respiratory system; those of the circulatory system; and symptoms, signs, and abnormal clinical findings not elsewhere classified. This finding is similar to that of a recent Danish study [8], with the exception for diseases of the circulatory system, which were more frequently noted in our study (23% versus 13%). Moreover, the three most commonly observed aetiologies were COPD (20%), pulmonary infection (17%), and heart failure (15%). In the United States, a lower frequency of COPD (13%) was observed [12], whereas a study from Australia/New Zealand showed higher rates of pulmonary infections (23%) and heart failure (20%) [11].

The patients in whom dyspnoea was the main symptom represent an older population (median age 77 years) with a high comorbidity. Such findings have also been reported in previous studies [8, 11, 12]. This is not unexpected since the incidence of chronic conditions associated with dyspnoea (e.g. COPD, heart failure, and coronary artery disease) increase with age [18]. Surprisingly, the majority had previously experienced dyspnoea. In our result, more than 60% of all patients were hospitalised. This is more than the entire EMS population in the same sampling area where the median age was 69 years and 50% of those taken to the ED were hospitalised [19].

Overall, 11% of our study patients died within 30 days, similar to previous studies [8, 9]. The mortality risk was almost three times as high as in the total EMS population in the same area [19]. Furthermore, our result also found that men with dyspnoea as the main symptom had a higher mortality rate than women. We have no clear explanation to this finding. There was no significant difference between women and men in terms of their final diagnosis with the exception of a lower rate of pulmonary disease among women. But men had a more severe comorbidity and more frequently suffered from a previous history of a cardiovascular disease and renal disease. This may have contributed the higher mortality among men. From an overall population perspective [20], it has been denoted that men have a greater vulnerability to cardiovascular diseases which is a reason to their higher mortality rate in general.

Patients attending EMS due to dyspnoea appear to have a five times higher risk of death than those with chest pain [9, 21]. This is in agreement with other reports saying that regardless of the aetiology, patients who have dyspnoea in combination with other conditions have a poor prognoses [22]. Thus, dyspnoea should be perceived as a high-risk symptom and a strong predictor of an increased mortality risk [9, 11, 23, 24]. Further, respiratory failure has been defined as among the five First Hour Quinted emergency time-related pathologies. This means that immediate prehospital treatment and early diagnosis may be crucial in reducing the morbidity and mortality risk [6].

This should be attributed to the fact that patients only call EMSs when they cannot manage the situation themselves and indeed half of patients waited more than 2 days after symptom onset in our study. In one previous study [25], patients tried to ignore their symptoms or manage them (working through), leading to delays in calling EMSs; some of these patients even took a “waiting approach”, while others were frustrated by their symptoms. This information is vital for consideration in the meeting between the PEN and patient.

The dyspnoea experience varies across people, and one can assume that some degree of tolerance to dyspnoea already existed in our patient group due to their comorbidities (e.g. COPD, heart failure, and hypertension). This is supported by earlier studies [10, 23] in which this patient group was characterised by poor health. Dyspnoea, to some extent, is a part of natural ageing and results in a decreasing degree of physical capacity; thus, it is not always explained by a specific illness or related to comorbidity presence. It may also be that elderly people are more tolerant and do not want to interfere or be perceived as disruptive. Thus, they may understate their symptom experience and severity when finally calling for help. This makes it even more difficult for both dispatchers and the PEN to determine disease severity [11].

Almost one in four patients had atrial fibrillation and a few had signs of myocardial ischemia on ECG, indicating that a cardiac pathology was not uncommon. This is most likely explained by the high degree of cardiovascular comorbidity reported in such populations [26–29]. Atrial fibrillation and acute heart failure are often seen in combination, and since heart failure is one of the dominating aetiologies behind dyspnoea, this was an expected finding. Based on previous history, one may assume that when atrial fibrillation occurred, it was most often previously known. Dyspnoea due to acute heart failure and the presence of ECG-related abnormalities, such as ventricular or supraventricular arrhythmia, bradycardia, or ongoing myocardial ischemia, may all be indicators of an increased risk of early hospital death [30].

One in four patients experienced pain, which may be important from a diagnostic as well as therapeutic perspective. The pain experience was mostly related to breathing-related chest discomfort, thoracic pain, chest wall pain, shoulder pain, or pain due to other unclear reasons. The presence of pain in dyspnoea patients has previously been reported [9, 21, 22, 31], mostly in the form of chest pain as a result of underlying causes including pulmonary embolism myocardial infarction, pneumonia and panic disorder.

A large proportion had hyperglycaemia. This may be an alarming sign also among patients with dyspnoea. Hyperglycaemia is a marker of acute stress response which, especially in nondiabetic patients, is associated with higher rates of in-hospital complications and mortality [15, 32].

Although only 11% had time critical final diagnoses, a much larger proportion of these patients will be regarded as having a time-critical condition in the acute phase since the majority had abnormal VS, primarily an abnormal respiratory rate and oxygen saturation. This requires advanced knowledge from the PENs who is caring for patients with dyspnoea, as abnormal vital parameters may be a sign of a number of different serious conditions; both cardiogenic, pulmonary and other aetiologies of acute respiratory failure. This highlights the importance of early recognition of the patients condition and directed treatment already at an early stage by EMS, as it might be crucial for the final outcome [33–35]. It has previously been reported that acute ill patients (including those with dyspnoea) admitted thorough ED [36] with abnormal vital parameters, have an increased risk of death. The most powerful predictors were abnormal respiratory rate, oxygen saturation and Glasgow Coma scale (GCS).

### Clinical implications

Our results suggest that 11% of all patients seen by PENs due to dyspnoea have time critical diagnosis and 11% will die during the subsequent 30 days. Available information from age, sex, comorbidity and clinical findings including vital parameters, ECG recordings, blood glucose measurements and other symptoms may form the basis for the building of future decision support tools in order to differentiate patients with high and low risk for future adverse events.

### Strengths and limitations

The major strength of our study is that data were collected from a relatively large representative sample. While the data were collected from a mix of urban and rural areas, the study itself is limited to a specific region of Sweden (southwest) which may impede the degree of generalisability of the findings to other settings. People living in the northern part of Sweden have greater difficulties in accessing EMSs and longer transport distances, which may lead to result variations.

Furthermore, due to the study design, the data had to be retrospectively collected from patient records, and important clinical parameters such as VS may have been measured but never recorded. In both EMS and hospital records, the documentation was sometimes insufficient. Likewise, it is possible that patients with other main symptoms who still have dyspnoea may have been classified into other ESS codes (not only ESS 04) by the PEN, as dyspnoea is also present in other conditions such as chest pain.

Information on the final diagnosis was missing in 1030 of all missions. Such cases were those left on- scene in whom there was no information on final diagnosis and patients who were brought to hospital and directly sent home from the Emergency Department, since some of them were never assessed by a physician. However, the present study was conducted in accordance with a paper by Kaji et al. [37], with the aim of reducing the bias associated with medical record reviews.

## Conclusions

Dyspnoea, as the main symptom, constituted approximately 9% of all the EMS missions in our study. A majority of the patients taken to hospital with dyspnoea as the main symptom were hospitalised and more than 400 different final diagnoses were observed, of which 11% were regarded as time-critical. A very high proportion (84%) had previously suffered from dyspnoea, which may explain why more than half showed delays greater than 2 days from symptom onset to EMS contact. High comorbidity values may contribute to this finding. Likewise, that patients call for help only when the situation is truly unmanageable makes them even more vulnerable. Dyspnoea, as the main symptom, is associated with a high risk of death, and 11% of all patients were dead within 30 days. However, more than half of these patients had abnormal vital parameters at the time of PEN assessment. This knowledge is important, not only for EMSs, but also for clinicians and those responsible for the provision of education in different emergency courses.

## Supplementary information

**Additional file 1.** The supplementary file describes in more detail deviating vital signs Red/Orange level according to RETTS-A (2017 version) and RETTS ESS 04 (main symptom of dyspnoea).

## Data Availability

The datasets analysed during the current study are available from the corresponding author on reasonable request.

## Abbreviations

COPD: Chronic obstructive pulmonary disease
ECG: Electrocardiogram
ED: Emergency department
EMS: Emergency medical service
ESS: Emergency signs and symptoms
ICD-10: International Statistical Classification of Diseases and Related Health Problems – 10th revision
PEN: Prehospital emergency nurse
RETTS-A: Rapid emergency triage and treatment system for adults
VS: Vital signs
