# Supplementary material for: Characteristics and outcomes of patients with dyspnoea as the main symptom, assessed by prehospital emergency nurses- a retrospective observational study
Source: BMC Emerg Med. 2020 Aug 28;20:67. doi: 10.1186/s12873-020-00363-6 (PMC7456019; doi:10.1186/s12873-020-00363-6)
Supplement: Supplementary file 1 — Additional file 1. The supplementary file describes in more detail deviating vital signs Red/Orange level according to RETTS-A (2017 version) and RETTS ESS 04 (main symptom of dyspnoea). [file 12873_2020_363_MOESM1_ESM.docx]

**Additional file 1.**

**Deviating vital signs Red/Orange level according to RETTS-A (2017 version)**

| A | Obstructive airway (Red level) | Threat to airway (Orange level) |
| --- | --- | --- |
| B | Respiratory rate > 30 /min  Respiratory rate < 8 /min  Oxygen saturation with supplemental oxygen < 90% | Respiratory rate > 25 /min  Oxygen saturation < 90% |
| C | Pulse rate > 130 /min  Irregular pulse rate > 160 /min  Systolic blood pressure < 90 mm/Hg | Pulse rate > 120 /min  Pulse rate < 40 /min |
| D | Ongoing seizures  RLS ≥ 4  GCS ≤ 9 | Somnolence  RLS 2-3  GCS 10-12 |
| E |  | Temperature < 35 or > 41 °C |

RLS: reaction level scale; GCS: Glasgow coma scale

Predicare AB (www.predicare.eu)

**RETTS ESS 04 (main symptom of dyspnoea)**

- Recent emerged left bundle branch block
- ST- elevation
- Sudden widespread thoracic pain, including vegetative symptoms or decreased consciousness
- Dyspnoea including ongoing chest pain with or without breathing correlation, and/or decreased consciousness
- Visible jugular vein stasis
- Ischemic signs on ECG + Dyspnoea

- Moderate/mild chest pain including normal ECG
- Risk factors
- With the absence of chest pain

**-** None of the above

**Assessment required in all triage levels: ECG**

ECG: Electrocardiogram

Predicare AB (www.predicare.eu)
